# Social Engagement of Caregivers: A Concept Analysis

**DOI:** 10.1093/geroni/igaf122.4275

**Published:** 2025-12-31

**Authors:** Chih Yu Wang, Hyeyeon Shin, Kathy Wright

**Affiliations:** The Ohio State University, Columbus, Ohio, United States; The Ohio State University, Columbus, Ohio, United States; The Ohio State University, Powell, Ohio, United States

## Abstract

Social engagement is critical for caregivers, as isolation increases their risk for depressive symptoms and anxiety. Social engagement is not well-defined and is used interchangeably with other terms. We aim to define social engagement by examining its attributes, antecedents, consequences, and empirical referents in caregivers. Following Walker and Avant’s concept analysis, five databases—PubMed, CINAHL, PsycINFO, Scopus, and Web of Science—were utilized (with no year limit). The Stress Process Model was applied, and the Social-Ecological Model mapped the multilevel contextual influences on caregiver social engagement. Two authors independently screened the abstracts and full texts. Quality was assessed using the Joanna Briggs Institute tool. Twenty-seven studies define social engagement as actively interacting with others in safe, supportive environments where caregivers build meaningful relationships over time.” Four attributes were identified: engagement behaviors in social activities, shared and supportive contexts for interaction, meaningful relationships, and level of interaction. Four antecedent categories appeared at all socioecological levels, except for the macrosystem, and consequences were grouped into benefits and risks. None of the studies used a validated instrument or captured all four attributes. Five studies assessed activity types, nine captured shared contexts, two addressed meaningful relationships, two measured engagement levels, six measured contact frequency, and three measured network size. By providing a more precise conceptual definition of social engagement, this review lays the groundwork for future research endeavors. Moving forward, developing validated measures and exploring contextual variations in caregiver social engagement are critical.

